# Nitric oxide attenuates overexpression of Giα proteins in vascular smooth muscle cells from SHR: Role of ROS and ROS-mediated signaling

**DOI:** 10.1371/journal.pone.0179301

**Published:** 2017-07-10

**Authors:** Oli Sarkar, Yuan Li, Madhu B. Anand-Srivastava

**Affiliations:** Department of Pharmacology and Physiology, Faculty of Medicine, University of Montréal, Montréal, Canada; Max Delbruck Centrum fur Molekulare Medizin Berlin Buch, GERMANY

## Abstract

Vascular smooth muscle cells (VSMC) from spontaneously hypertensive rats (SHR) exhibit decreased levels of nitric oxide (NO) that may be responsible for the overexpression of Giα proteins that has been shown as a contributing factor for the pathogenesis of hypertension in SHR. The present study was undertaken to investigate if increasing the intracellular levels of NO by NO donor S-Nitroso-N-acetyl-DL-penicillamine (SNAP) could attenuate the enhanced expression of Giα proteins in VSMC from SHR and explore the underlying mechanisms responsible for this response. The expression of Giα proteins and phosphorylation of ERK1/2, growth factor receptors and c-Src was determined by Western blotting using specific antibodies. Treatment of VSMC from SHR with SNAP for 24 hrs decreased the enhanced expression of Giα-2 and Giα-3 proteins and hyperproliferation that was not reversed by 1H (1, 2, 4) oxadiazole (4, 3-a) quinoxalin-1-one (ODQ), an inhibitor of soluble guanylyl cyclase, however, PD98059, a MEK inhibitor restored the SNAP-induced decreased expression of Giα proteins towards control levels. In addition, the increased production of superoxide anion, NAD(P)H oxidase activity, overexpression of AT1 receptor, Nox4, p22^phox^ and p47^phox^ proteins, enhanced levels of TBARS and protein carbonyl, increased phosphorylation of PDGF-R, EGF-R, c-Src and ERK1/2 in VSMC from SHR were all decreased to control levels by SNAP treatment. These results suggest that NO decreased the enhanced expression of Giα-2/3 proteins and hyperproliferation of VSMC from SHR by cGMP-independent mechanism and involves ROS and ROS-mediated transactivation of EGF-R/PDGF-R and MAP kinase signaling pathways.

## Introduction

Heterotrimeric guanine nucleotide-binding regulatory proteins (G proteins) have been shown to activate various signal transduction systems, including the adenylyl cyclase/cAMP system that regulate of a variety of physiological functions including blood pressure [1]. The hormonal stimulation and inhibition of adenylyl cyclase are mediated by two G proteins known as stimulatory (Gs) and inhibitory (Gi) respectively and are composed of α, β and γ subunits [2–4]. Four different isoforms of Gsα proteins resulting from the differential splicing of a single gene [5, 6] and three isoforms of Giα proteins, Giα-1,2 and 3, products of three distinct genes [7] have been identified by molecular cloning. All the three isoforms of Giα proteins mediate the adenylyl cyclase inhibition and atrial K^+^ channels activation [7, 8]

Several cellular functions including vascular tone, cell proliferation etc, that are implicated in the regulation of blood pressure are mediated through the activation of Giα proteins and associated adenylyl cyclase signaling [9–12]. Alterations in the levels of Giα-2 and Giα-3 proteins lead to various pathological states including hypertension. An increased expression of Giα-2 and Giα-3 proteins and their mRNA in cardiovascular tissues from spontaneously hypertensive rats (SHR) [13–15], deoxycorticosterone acetate (DOCA)-salt [16], L-NAME [17] and 1-Kidney-1Clip [18] hypertensive rats has been reported. The increased expression of Giα-2 and Giα-3 proteins and resultant decreased levels of cAMP were shown to contribute to the pathogenesis of hypertension in spontaneously hypertensive rats (SHR) and DOCA-salt hypertensive rats [19, 20]. This was further supported by the studies showing that the inactivation of Giα proteins in prehypertensive rats (2 weeks old SHR) by single injection of pertussis toxin (PT) prevented the development of high blood pressure that was associated with PT-induced decreased levels of Giα proteins [21]. Furthermore, the increased levels of endogenous angiotensin II (Ang II) and ET-1 exhibited by VSMC from SHR [22, 23] were shown to enhance the expression of Giα-2 and Giα-3 proteins through reactive oxygen species (ROS)-mediated c-Src and transactvation of growth factor receptors and MAP kinase signaling pathways [24, 25]. A role of enhanced expression of Giα-2 and Giα-3 proteins has also been shown in hyperproliferation of vascular smooth muscle cells (VSMC) [11, 12, 26] that contributes to vascular remodeling associated with hypertension [27].

Nitric oxide (NO) is a diffusible messenger that plays a role in a variety of physiological functions including vasorelaxation, inhibition of platelet aggregation, inflammation, neurotransmission, hormone release, cell differentiation, migration, and apoptosis [28, 29]. Most of the effects have been shown to be mediated through the activation of soluble guanylyl cyclase and cGMP pathways [30]; however, other cGMP-independent mechanisms have also been reported [29, 31]. We earlier showed that the inhibition of NO-synthase by N^ω^-nitro-L-arginine methyl ester (L-NAME) treatment of rats that decreases the levels of intracellular NO, results in the enhanced expression of Giα-2 and Giα-3 proteins and augmentation of blood pressure [17]. Furthermore, the decreased levels of NO and eNOS have been shown in SHR [32, 33] which may be responsible for the enhanced expression of Giα proteins and resultant high blood pressure. The present study was undertaken to investigate if the augmentation of intracellular levels of NO by NO donor, SNAP could attenuate the enhanced expression of Giα-2 and Giα-3 proteins and hyperproliferation of VSMC from SHR and to explore the underlying molecular mechanisms responsible for this response.

We provide evidence that SNAP decreased the enhanced expression of Giα proteins and hyperproliferation of VSMC from SHR by a cGMP-independent mechanism. The decreased expression of Giα-2 and Giα-3 proteins induced by NO occurs through its ability to attenuate the enhanced oxidative stress, activation of growth factor receptors and MAP kinase signaling. Thus, it is suggested that NO-mediated decreased expression of Giα-2 and Giα-3 proteins may be another mechanism through which NO regulates blood pressure.

## Materials and methods

### Materials

S-Nitroso-N-acetyl-DL-penicillamine (SNAP), 1H (1, 2, 4) oxadiazole (4, 3-a) quinoxalin-1-one (ODQ), 8-Bromoguanosine 3`,5`-cyclicmonophosphate (8-Br-cGMP) and PD98059 were purchased from Sigma-Aldrich Chemical Co. (St Louis, Missouri, USA). Antibodies against AT1 receptor (N-10), G_i_α-2 (L5), G_i_α-3 (C-10), ERK1/2 (C-14), p-ERK1/2 (phosphospecific-tyrosine^204^), p-AKT (phosphospecific-serine^473^), AKT (H-136), p-PDGF-R (phosphospecific-tyrosine^857^), PDGF-R (958), p-EGF-R (phosphospecific-tyrosine^1173^), EGF-R (1005) and (phospho)-c-Src (phosphospecific-tyrosine-419), monoclonal dynein IC1/2 antibody (74–1), monoclonal anti-β-actin antibody (A5441), p22^phox^, p47^phox^ (C-17), Nox4 (N-15), and Western blotting reagents were from Santa-Cruz Biotechnologies (Santa Cruz, CA, USA). All other chemicals used in the experiments were purchased from Sigma-Aldrich. Male spontaneously hypertensive rats (SHR) and age-matched Wistar Kyoto rats (WKY) were purchased from Charles River (St-Constant, Quebec, Canada).

### Cell Culture and Incubation

12-week-old male spontaneously hypertensive rats (SHR) and age-matched Wistar Kyoto (WKY) rats were euthanized by decapitation. The aorta were dissected out and VSMC from SHR and WKY rats were cultured as described previously [34, 35]. The purity of the cells was checked by immunofluorescence technique using α-actin as described previously [36]. These cells were found to contain high levels of smooth muscle-specific actin. The cells were plated in 75-cm^2^ flasks and incubated at 37°C in 95% air-5% CO_2_ humidified atmosphere in DMEM (with glucose, L-glutamine, and sodium bicarbonate) containing antibiotics and 10% heat-inactivated FBS. The cells were passaged upon reaching confluence with 0.5% trypsin containing 0.2% EDTA and utilized between passages 4 and 12. Subconfluent cells were then starved by incubation for 3 h in DMEM without FBS at 37°C to reduce the interference by growth factors present in the serum. To examine the effect of ODQ or PD98059 on SNAP-induced decreased expression of Giα proteins, the cells were preincubated in the absence (control) or presence of ODQ (20 μM), or PD98059 (10 μM) for 30 mins prior to the treatment with SNAP (100 μM) for 24 h. This time of treatment with SNAP has been shown to decrease the expression of Giα proteins and natriuretic peptide receptor-C in VSMC [37, 38] and suggests that SNAP at 24 h of treatment is stable and active. After incubation, the cells were washed twice with ice-cold phosphate-buffered saline (PBS) and lysed in a 200 μl buffer containing 25 mM Tris·HCl (pH 7.5), 25 mM NaCl, 1 mM sodium orthovanadate, 10 mM sodium fluoride, 10 mM sodium pyrophosphate, 2 mM EDTA, 1 mM phenylmethylsulfonyl fluoride, 10 μg/ml aprotinin, 1% Triton X-100, 0.1% sodium dodecyl sulfate, and 0.5 μg/ml leupeptin on ice. The cell lysates were centrifuged at 12,000 rpm for 10 min at 4°C, and the supernatants were used for Western blot analysis using specific antibodies. Cell viability was checked by the trypan blue exclusion technique and indicated that >90~95% cells were viable

All the animal procedures used in the present studies were approved by the Comité de Déontologie de l'Expérimentation sur les Animaux (CDEA) of the University of Montreal (#99050). The investigation conforms to the 'Guide for the Care and Use of Laboratory Animals published by the US National Institutes of Health (NIH) (Guide, NRC 2011).

### Western blotting

Western blotting of Gi proteins, Nox4, p47phox, phosphorylated and unphosphorylated c-Src, ERK 1/2, PDGFR, EGFR, were performed using specific antibodies as described previously [10, 34, 39, 40]. Following separation on SDS-PAGE, proteins were electrophoretically transferred to nitrocellulose paper with a semi-dry transblot apparatus (Bio-Rad Laboratories, Mississauga, Ontario) at 15 V for 45 min. After transfer, the membranes were washed twice in phosphate-buffered saline (PBS) and were incubated in 5% dehydrated milk (w/v in PBS) at room temperature for one hour. The blots were washed with PBS containing 0.1% Tween-20 (PBST) and then incubated with respective primary antibodies against AT1(sc-1173), Giα-2 (sc-13534), Giα-3 (sc-262), Nox4 (sc-21860), p47^phox^ (sc-17845), p22^phox^ (sc-11712), PDGFR (sc-432), p-PDGFR (sc-12907) EGFR (sc-03), p-EGFR (sc-12351) using different dilutions ranging from 1:500 to 1:2000 at room temperature for 2 h. The antigen-antibody complexes were detected by incubating the blots with goat anti-rabbit IgG (Bio-Rad) conjugated with horseradish peroxidase for 1 h at room temperature. The blots were then washed three times with PBS before reaction with enhanced chemiluminescence Western blotting detection reagents (Santa Cruz, CA, USA). Blots were reprobed with Dynein (sc-13524) or β-actin (Sigma, A-5441) as loading controls. Quantitative analysis of the protein was performed by densitometric scanning of the autoradiographs employing the Enhanced Laser Densitometer LKB Ultroscan XL and quantified using the gel-scan XL evaluation software (version 2.1) from Pharmacia (Baie d'Urfé, Québec, Canada). The scanning was one dimensional and scanned the entire area of protein bands in the blot.

### Determination of superoxide anion production

Basal superoxide anion production and NADPH oxidase activity in VSMC was measured using the lucigenin-enhanced chemiluminescence method with low concentration (5 μmol/l) of lucigenin as described previously [34, 41]. VSMC from control and SNAP-treated SHR and WKY rats were washed in oxygenated Kreb—Hepes buffer, and placed in scintillation vials containing lucigenin solution, and the emitted luminescence was measured with a liquid scintillation counter (Wallac 1409; Perkin Elmer Life Science, St Laurent, Quebec, Canada) for 5 min. The average luminescence value was estimated, the background value subtracted and the result was divided by the concentration of proteins in each sample.

### Determination of NAD(P)H oxidase activity

After the emitted luminescence for basal superoxide anion production was measured, 0.1 mM NADPH was added in the vials and the luminescence was measured continuously for 5 min in a liquid scintillation counter (Wallac 1409; PerkinElmer Life Science). NAD(P)H oxidase activity was calculated by subtracting the basal superoxide-induced luminescence from the luminescence value induced by NADH.

### Determination of Thiobarbituric acid-reactive substances (TBARS)

Lipid peroxidation was determined by measuring TBARS in control and SNAP-treated cells as described earlier [42] Cells were preincubated with 0.01 mM CuCl_2_ in 20 mM phosphate buffer (pH 7.4) at room temperature for 15 min. The reaction was started by the addition of 0.5 mM ascorbate and 1.9 mM deoxyribose and incubated for 1 h at 37°C. Thiobarbituric acid (10 g/L) in 50 mM NaOH and concentrated acetate acid (1:1 ratio) were added to the incubation mixture, which was boiled in water for 15 min. The TBARS were quantified by spectrophotometer at 532 nm. Phosphate buffer (20 mM; pH 7.4) was taken as blank. Protein concentration was measured with a Bio-Rad (Hercules, CA, USA) assay kit using bovine serum albumin as a standard.

### Determination of protein carbonyl

The carbonyl content of proteins in control and SNAP-treated cells was determined by the 2,4-dinitrophenylhydrazine (DNPH) method as described earlier [43]. Cells were lysed and proteins were precipitated with 20% trichloroacetic acid. The precipitates were incubated with either 2 N HCl alone (blank) or 2 N HCl containing 10 mM DNPH at room temperature in the dark for 1 h, being vortexed every 10 min. After the reaction, the mixture was centrifuged, and the precipitates were washed with an ethanol:ethyl acetate (1:1) mixture three times and dissolved in 6 M guanidine chloride or 8 M urea. The absorbance was measured at 360 nm. The concentration of protein was measured with a Bio-Rad assay kit using bovine serum albumin as a standard.

### Cyclic GMP level measurement

Cyclic GMP (cGMP) levels were quantitatively measured by using an ELISA (enzyme-linked immunosorbent assay) kit (Abcam, 330 Cambridge Science Park, Cambridge, CB40FL, UK) according to the manufacturer's instructions as described previously [44]. and the concentration was expressed as picomoles per mg of protein. All the ELISA experiments were repeated at least four times.

### Determination of intracellular nitric oxide (NO)

The levels of intracellular NO in VSMC were measured using intracellular fluorescent probes. Confluent VSMC, after washing twice with phosphate buffer solution (PBS) were incubated at 37°C for 1 h with both 10^−2^ mol/L diaminofluorescein-2 diacetate (DAF-2 DA) and 10^−6^ mol/L acetylcholine for detecting NO. Cells were washed twice with PBS and fluorescence intensities were measured by a spectrophotometer (TECAN infinite 200 PRO) with excitation and emission wavelengths at 495 nm and 515 nm for DAF-2DA, Changes in fluorescence intensities were expressed as percentages of the values obtained in the WKY group (taken as 100%).

### Determination of cell proliferation

Cell proliferation was quantified by DNA synthesis that was evaluated by incorporation of [^3^H] thymidine into cells as described earlier [9]. Subconfluent aortic VSMC from SHR and WKY rats were plated in six-well plates for 24 h and were serum deprived for 24 h to induce cell quiescence. VSMC were incubated in the absence or presence of SNAP (100 μM) for 24 h. [^3^H] thymidine (1 μCi/ml) (PerkinElmer Life Science) was added and further incubated for 4 h before the cells were harvested. The cells were rinsed twice with ice-cold PBS and incubated with 5% trichloroacetic acid for 1 h at 4°C. After being washed twice with ice-cold water, the cells were incubated with 0.4 N sodium hydroxide solution for 30 min at room temperature, and radioactivity was determined by liquid scintillation counter. Cell viability was checked by the trypan blue exclusion technique and indicated that >90∼95% cells were viable.

### Data presentation and statistical analysis

The number of independent experiments is reported. Each experiment was conducted at least three times using separate cell population. All data are expressed as the mean ± SD. Comparisons between groups were made with one way analysis of variance (ANOVA) followed by Dunnett tests using GraphPad Prism5 software. Results were considered significant at a value of p < 0.05.

## Results

### NO donors attenuate the enhanced expression of Giα protein in VSMC from SHR

**Fig 1:**
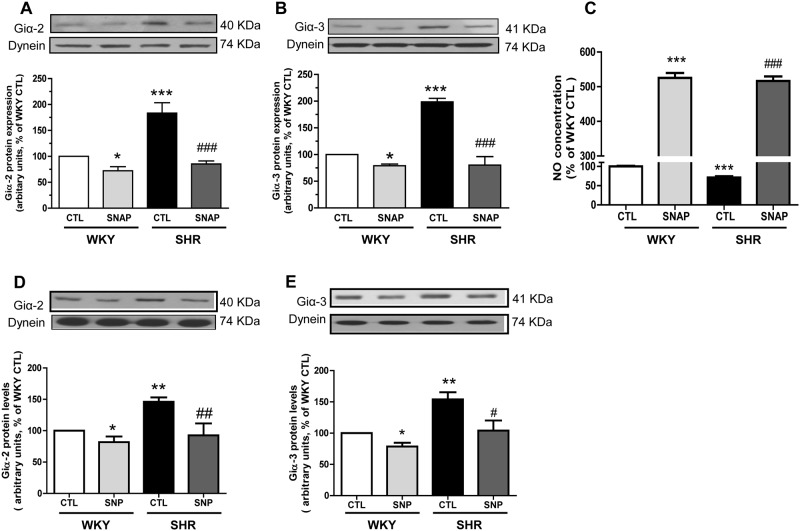
**(A and B) Effect of S-nitroso-N-acetyl penicillamine (SNAP) and sodium nitroprusside (SNP) (D and E) on the Giα protein expression in the aortic vascular smooth muscle cells (VSMC) from 12-week old spontaneously hypertensive rats (SHRs) and age-matched Wistar-Kyoto (WKY) rats.** Confluent VSMCs were starved 24 h and incubated in the absence (control) or presence of SNAP (100 μM) (A and B) or SNP (100 μM) (D and E) for 24 h. The cell lysates were prepared and subjected to Western blot analysis using specific antibodies against Giα-2 (A and D) and Giα-3 (B and E) or dynein as loading control, (upper panel) as described in the Materials and methods. **(C): Effect of SNAP treatment on NO production in aortic vascular smooth muscle cells (VSMC) from 12-week old spontaneously hypertensive rats (SHRs) and age-matched Wistar-Kyoto (WKY) rats.** Confluent VSMC from SHR and WKY rats with or without SNAP treatment were incubated at 37°C for 1 h with fluorescent probes diaminofluorescein-2 diacetate (DAF-2DA) and levels of NO were determined as described in Materials and Methods. Results are expressed as percentages of WKY control group (taken as 100%). Values are mean ± SD of 4 separate experiments using different cell populations from different animals. *P<0.05, **P<0.01 ***P<0.001 vs WKY control (CTL); ^#^P<0.05, ^##^P<0.01, ^###^P<0.001 vs SHR CTL.

SHR have been shown to exhibit decreased levels of NO and eNOS [32, 33] which may be responsible for the overexpression of Giα proteins in SHR. To investigate this, we examined the effect of augmenting the levels of intracellular NO by SNAP and sodium nitroprusside (SNP) on the expression of Giα-2 and Giα-3 proteins in VSMC from SHR and WKY rats and the results are shown in “Fig 1“. As reported earlier (13), the expression of Giα-2 protein (A) and Giα-3 (B) was increased by ~80% and 100% respectively in VSMC from SHR as compared to WKY rats and the treatment of cells with SNAP for 24 h that increases the levels of NO by about 630% and 450% respectively (C) in VSMC from SHR and WKY rats completely attenuated the enhanced expression of Giα proteins to WKY levels. In addition, SNP, another donor of NO also attenuated the enhanced expression of Giα-2 (D) and Giα-3 (E) by about 90% and 80% respectively in VSMC from SHR whereas the expression of Giα-2 and Giα-3 in VSMC from WKY rats was also decreased by both SNAP and SNP by 30% and 20% respectively.

**Fig 2:**
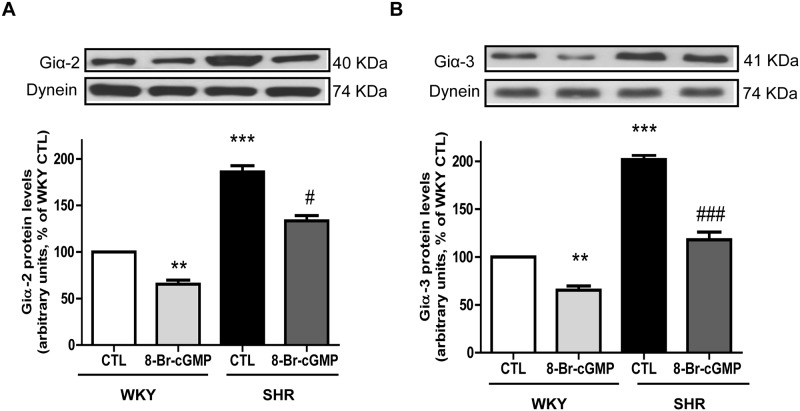
The effect of 8-Br-cGMP on the Giα protein expression in the aortic vascular smooth muscle cells (VSMC) from 12-week old spontaneously hypertensive rats (SHRs) and age-matched Wistar-Kyoto (WKY) rats. Confluent VSMCs were starved 24 h and incubated in the absence (control) or presence of or 8-Br-cGMP (0.5mM) for 24 h. The cell lysates were prepared and subjected to Western blot analysis using specific antibodies against Giα-2 (A) and Giα-3 (B)) or dynein as loading control, (upper panel) as described in the Materials and methods. Quantification of protein bands was done by densitometric scanning (lower panel). The results are expressed as a percentage of the WKY control (CTL), taken as 100%. Values are the mean ± SD of 4 separate experiments using different cell populations from different animals. **P<0.01, *** P<0.001 vs WKY CTL group, ^#^P<0.05, ^###^ P<0.001 vs SHR CTL group.

Since NO activates the soluble guanylyl cyclase activity and augments the levels of intracellular cGMP which has been shown to inhibit the expression of Giα proteins in aortic and A10 VSMC [45, 46], it was desirable to examine if cGMP could also attenuate the enhanced expression of Giα proteins in VSMC from SHR. Results shown in “Fig 2A and 2B” indicate that 8-Br-cGMP, a cell permeable analog of cGMP (0.5 mM) also inhibited the enhanced expression of Giα-2 (A) and Giα-3 (B) proteins in VSMC from SHR by about 60 and 90% respectively whereas about 35% inhibition in the expression of these proteins was observed in VSMC from WKY rats. Furthermore, 8-Br-cGMP, at concentrations below 0.5 mM was ineffective in attenuating the enhanced expression of Giα-2 and Giα-3 proteins in VSMC from SHR.

### Effect of ODQ on SNAP-induced decreased expression of Giα proteins in VSMC from SHR

**Fig 3:**
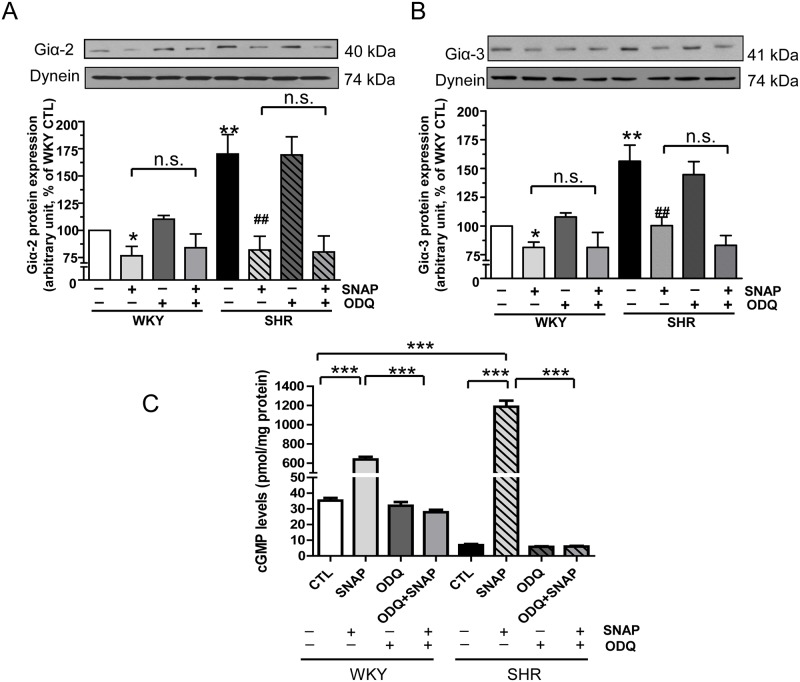
**(A and B) Effect of ODQ on SNAP- induced decreased expression of Giα proteins (A and B) in aortic vascular smooth muscle cells (VSMCs) from 12-week old SHR and age-matched WKY rats.** Confluent VSMC were incubated in the absence (control) or presence of 1H-(1, 2, 4) oxadiazolo [4, 3-a] quinoxalin-1-one (ODQ), for 30 mins prior to the treatment with SNAP for 24 h. The cell lysates were prepared and subjected to Western blot analysis using specific antibodies against Giα-2 (A) and Giα-3 (B) or dynein as loading control upper panel) as described in the Materials and methods. Quantification of protein bands was done by densitometric scanning (lower panel). The results are expressed as a percentage of the WKY control (CTL), taken as 100%. Values are the mean ± SD of 4 separate experiments using different cell populations from different animals. *P<0.05, ** P<0.01 vs WKY CTL group; ^##^ P < 0.01 vs SHR CTL group. NS, Not significant. (C) **Effect of ODQ on SNAP- induced increased levels of cGMP in aortic vascular smooth muscle cells (VSMCs) from 12-week old SHR and age-matched WKY rats.** Confluent VSMC were incubated in the absence (control) or presence of 1H-(1, 2, 4) oxadiazolo [4, 3-a] quinoxalin-1-one (ODQ), for 30 mins prior to the treatment with SNAP for 24 h. The levels of cGMP were determined by ELISA as described in the Materials and methods section. The concentration of cGMP levels were expressed as picomoles per mg of protein. Values are mean ± SD of 6 independent experiments using different cell populations from different animals. The results are expressed as percentage of WKY (control) which has been taken as 100%. ***P<0.001.

NO has been shown to mediate most of its effects through the activation of soluble guanylyl cyclase and cGMP pathways [30]. To further investigate if SNAP-induced decreased expression of Giα proteins is mediated through the activation of soluble guanylyl cyclase, we examined the effect of ODQ, an inhibitor of soluble guanylyl cyclase on the expression of Giα proteins and intracellular levels of cGMP in VSMC from SHR and WKY rats in the absence and presence of SNAP. As shown in “Fig 3“, SNAP decreased the expression of Giα-2 (A) and Giα-3 (B) in VSMC from SHR and WKY rats, however, inhibition of soluble guanylyl cyclase by pretreatment with ODQ did not affect the SNAP-induced decreased expression of Giα-2 or Giα-3 proteins. On the other hand, SNAP-induced increased levels of cGMP in VSMC from both SHR and WKY rats were completely abolished by ODQ (C). These results suggest that NO decreased the levels of Giα proteins by a cGMP-independent mechanism.

### SNAP attenuates the enhanced expression of AT1 receptor in VSMC from SHR

**Fig 4:**
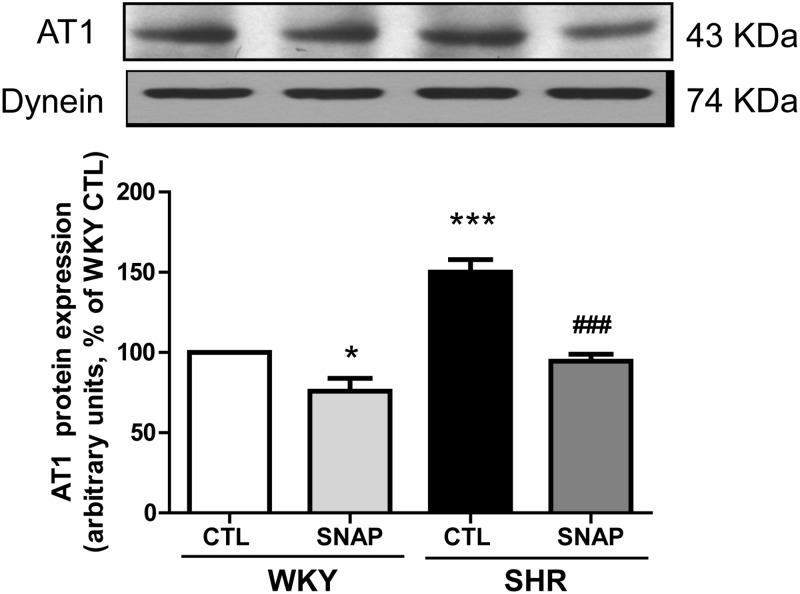
Effect of SNAP on the AT1 receptor expression in the aortic vascular smooth muscle cells (VSMC) from 12-week old spontaneously hypertensive rats (SHRs) and age-matched Wistar-Kyoto (WKY) rats. Confluent VSMCs were starved 24 h and incubated in the absence (control) or presence of SNAP (100 μM) for 24 h. The cell lysates were prepared and subjected to Western blot analysis using specific antibodies against AT1 (N-10) or dynein as loading control, (upper panel) as described in the Materials and methods. Quantification of protein bands was done by densitometric scanning (lower panel). The results are expressed as a percentage of the WKY control (CTL), taken as 100%. Values are the mean ± SD of 4 separate experiments using different cell populations from different animals.* P<0.05, *** P<0.001 vs WKY CTL group, ^###^ P<0.001 vs SHR CTL group.

Since the enhanced levels of endogenous angiotensin II (Ang II) through the activation of AT1 receptors were shown to contribute to the enhanced expression of Giα proteins in VSMC from SHR [24], it was of interest to examine if SNAP-induced attenuation of enhanced expression of Giα proteins is also due to its ability to inhibit the enhanced levels of AT1 receptor. To test this, we examined the effect of SNAP on the expression of AT1 receptor in VSMC from SHR and WKY rats and the results are shown in “Fig 4“. The expression of AT1 receptor is significantly augmented by about 50% in VSMC from SHR as compared to WKY rats and was restored to WKY control level by SNAP treatment. In addition, SNAP also decreased the expression of AT1 receptor by about 20% in WKY rats.

### SNAP attenuates enhanced oxidative stress in VSMC from SHR

**Fig 5:**
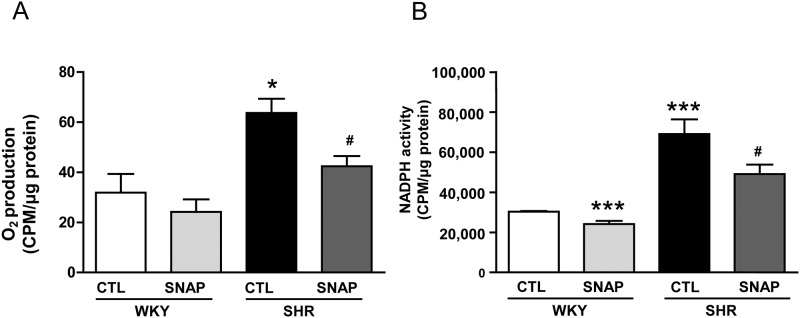
Effect of SNAP on superoxide anion (O2^−^) production and NADPH oxidase activity in VSMC from 12-week old SHR and age-matched WKY rats. Confluent VSMC from 12-week-old SHR and age-matched WKY rats were incubated in the absence (control) and presence of SNAP (100 μM) for 24 h and O_2_^−^ production (A) and NADPH oxidase activity (B) were determined as described in Materials and methods. Values are mean ± SD of 5 separate experiments using different cell populations from different animals. *P<0.05, ***P<0.001 vs WKY CTL group; ^#^P<0.05 vs SHR CTL group.

The enhanced oxidative stress was shown to contribute to the enhanced expression of Giα proteins in VSMC from SHR [24, 34]. To investigate if SNAP-induced attenuation of Giα protein levels is attributed to its ability to decrease the oxidative stress, the effect of SNAP on the production of superoxide anion (O_2_^-^) was examined in VSMC from SHR and WKY rats and the results are shown in “Fig 5“. As reported earlier [34, 47], the levels of O_2_^-^ (A) and NADPH oxidase activity (B) were significantly enhanced in VSMC from SHR by about 100% as compared to WKY rats and SNAP treatment attenuated the enhanced levels of O_2_^-^ and NADPH oxidase activity by about 50% and 35% respectively. In addition, SNAP also decreased O_2_^-^ production and NADPH oxidase activity in VSMC from WKY rats by 20% in WKY rats.

### SNAP attenuates the enhanced expression of NADPH oxidase subunits in VSMC from SHR

**Fig 6:**
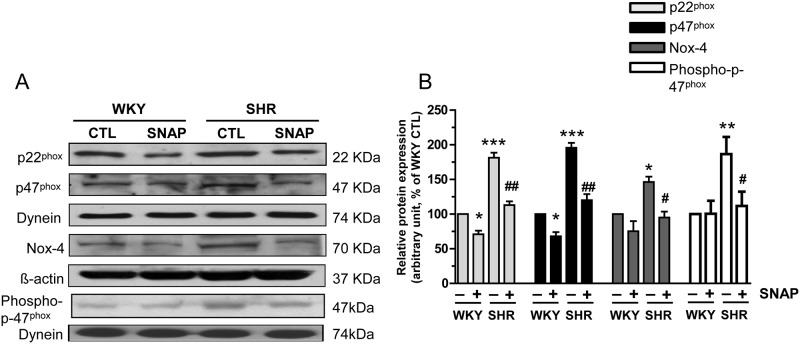
Effect of SNAP on the expression of NADPH oxidase subunits in aortic VSMC from 12-week old SHRs and age-matched WKY rats. Confluent VSMC were incubated in the absence (control) or presence of SNAP (100 μM) for 24 h. The cell lysates were prepared and subjected to Western blot analysis using specific antibodies against NADPH oxidase subunits p22phox, p47phox, phospho- p47phox and Nox-4 (A) as described in Materials and methods. Dynein was used as a loading control for p22phox, p47phox and phospho-p47phox whereas β-actin was used as loading control for Nox-4. Quantification of protein bands was done by densitometric scanning (B). The results are expressed as a percentage of the WKY control (CTL), taken as 100%. Values are the mean ± SD of 4 separate experiments using different cell populations from different animals. * P<0.05, **P<0.01, ***P< 0.01 vs WKY CTL group; ^#^ P < 0.05, ## P < 0.01 vs SHR CTL group.

To examine whether SNAP—induced decreased expression of different subunits of NADPH oxidase contributes to decreased activity of NADPH oxidase activity in VSMC from SHR, the effect of SNAP treatment on the expression of p22^phox^, p47^phox^ and Nox-4, critical subunits involved in NADPH oxidase activation was examined in VSMC from SHR and WKY rats. Results shown in “Fig 6“indicate that the levels of p22^phox^, p47^phox^, phospho- p47^phox^ and Nox-4 that were significantly enhanced in VSMC from SHRs by about 80%, 90%, 40% and 90% respectively as compared with WKY rats, were attenuated to control WKY level by SNAP treatment. In addition, SNAP treatment also decreased the levels of p22^phox^, p47^phox^ and Nox-4 by about 25% in WKY rats.

### SNAP attenuates enhanced TBARS levels and protein carbonylation in VSMC from SHR

**Fig 7:**
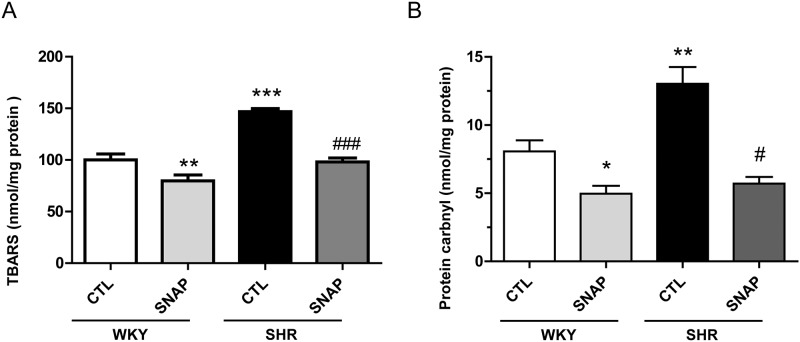
Effect of SNAP in TBARS and protein carbonylation in aortic VSMC from 12-week old SHR and age-matched WKY rats. Confluent VSMC from 12-week-old SHR and age-matched WKY rats were incubated in the absence (control) or presence of SNAP (100 μM) for 24 h. Cell lysates were prepared and the levels of TBARS (A) or protein carbonyls (B) were determined as described under Materials and methods Results were expressed as percentage of the WKY control (CTL), which was taken as 100%. Values are the Mean ± SD of 3 separate experiments using different cell populations from different animals. * P<0.05, ** P<0.01, *** P<0.001vs WKY CTL group; ^#^P<0.05, ^###^ P<0.001 vs SHR CTL group.

We also examined the effect of SNAP on the levels of TBARS as well as protein carbonyls, the markers of oxidative stress in VSMCs from SHR and the results are shown in “Fig 7“. The levels of TBARS (A) as well as protein carbonyls (B) were elevated by about 60% in VSMC from SHR compared to WKY and this increase was completely reversed by SNAP treatment. On the other hand, SNAP also decreased the levels of TBARS and protein carbonyls by 25% in WKY.

### Role of MAP Kinase in SNAP-induced attenuation of enhanced Giα protein expression in VSMC from SHR

**Fig 8:**
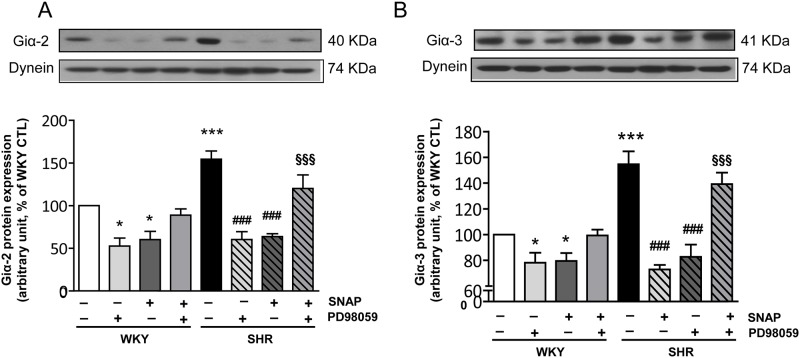
Effects of PD98059 in SNAP- induced decreased expression of Giα-2 and Giα-3 proteins in aortic VSMC from 12-week old SHR and age-matched WKY rats. Confluent VSMC were incubated in the absence (control) or presence of PD98059 (10 μM), for 30 mins prior to the treatment with SNAP for 24 h. The cell lysates were prepared and subjected to Western blot analysis using specific antibodies against Giα-2 (A) and Giα-3 (B) or dynein as loading control (upper panel) as described in the Materials and methods. Quantification of protein bands was done by densitometric scanning (lower panel). The results are expressed as a percentage of the WKY control (CTL), which was taken as 100%. Values are the mean ± SD of 5 separate experiments using different cell populations from different animals. * P<0.05, *** P<0.001 vs WKY CTL group; ^###^ P < 0.001 vs SHR CTL group; ^§§§^ P<0.001 vs SHR-SNAP treated group.

We earlier showed that the enhanced expression of Giα proteins in VSMC from SHR was attributed to the enhanced activity of MAP kinase [25, 26, 34]. In addition, NO has been shown to regulate MAPK activity [38, 48, 49]. To investigate the role of MAP kinase in SNAP-induced attenuation of enhanced expression of Giα proteins in VSMC from SHR, we inhibited MAP kinase activity by pretreating the cells with PD98059, a MEK inhibitor and then examined the effect of inhibition of MAP kinase on SNAP- induced decreased expression of Giα protein in VSMC from SHR and WKY rats. The results shown in “Fig 8“illustrate that SNAP and PD98059 alone inhibited the expression of Giα-2 (A) and Giα-3 proteins (B) in VSMC from SHR and WKY rats, however, SNAP was unable to decrease the expression of Giα-2 and Giα-3 proteins in cells pretreated with PD98059 suggesting the implication of MAPK in SNAP-induced decreased levels of Giα proteins.

**Fig 9:**
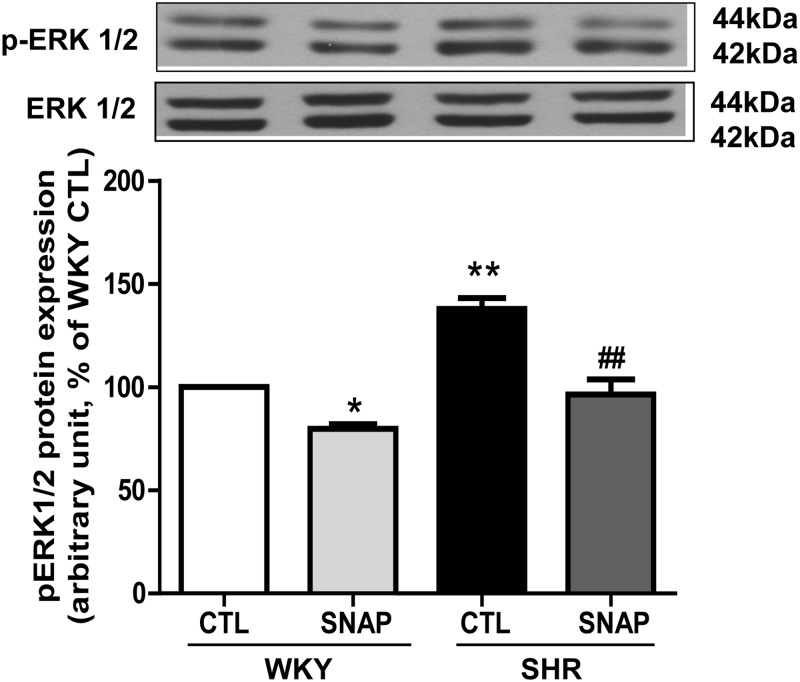
Effect of SNAP on ERK phosphorylation in VSMC from 12-week old SHR and age-matched WKY rats. Confluent VSMC were incubated in the absence (control) or presence of SNAP (100 μM) for 24 h. The cell lysates were prepared and subjected to Western blot analysis using specific antibodies against phosphorylated ERK1/2 (upper panels) as described in the Materials and methods. Quantification of protein bands was done by densitometric scanning (lower panels). The results are expressed as a percentage of the WKY control (CTL), taken as 100%. Values are the mean ± SD of 4 separate experiments using different cell populations from different animals. * P<0.05, ** P<0.01 vs WKY CTL group, ^##^ P<0.001 vs SHR CTL group.

To further investigate if SNAP could also attenuate the MAP kinase activation in VSMC from SHR, we examined the effect of SNAP on ERK1/2 phosphorylation in VSMC from SHR and the results are shown in “Fig 9“. As reported earlier [34, 38], the phosphorylation of ERK1/2 was significantly augmented by about 40% in VSMC from SHR compared with WKY and SNAP treatment attenuated the enhanced phosphorylation of ERK1/2 to control levels. In addition, this treatment also attenuated the ERK1/2 phosphorylation by about 25% in VSMC from WKY rats. Furthermore, the total ERK1/2 was unaltered in control and SNAP-treated VSMC from SHR and WKY rats.

### SNAP decreases growth factor receptor activation in VSMC from SHR

**Fig 10:**
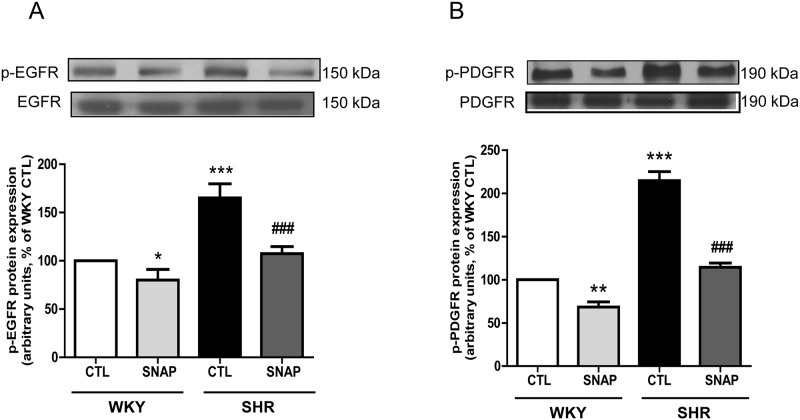
Effect of SNAP on EGFR phosphorylation and PDGFR phosphorylation protein levels in VSMC from 12-week old SHR and age-matched WKY rats. Confluent VSMC were incubated in the absence (control) or presence of SNAP (100 μM) for 24 h. The cell lysates were prepared and subjected to Western blot analysis using specific antibodies against phosphorylated EGFR (A) or phosphorylated PDGFR (B, upper panels) as described in the Materials and methods. Quantification of protein bands was done by densitometric scanning (Lower panels). The results are expressed as a percentage of the WKY control (CTL), which was taken as 100%. Values are the mean ± SD of 6 separate experiments using different cell populations from different animals. * P<0.05, **P<0.01, *** P<0.001 vs WKY CTL group, ^###^ P<0.001 vs SHR CTL group.

Since the enhanced expression of Giα proteins in VSMC from SHR was shown to be attributed to the transactivation of EGF-R and PDGF-R due to the enhanced levels of endogenous vasoactive peptides [24, 25], it was of interest to examine if SNAP-induced attenuation of Giα proteins in VSMC from SHR is mediated through the inhibition of EGF-R and PDGF-R activation. To test this, the effects of SNAP on the phosphorylation of EGF-R and PDGF-R were examined in VSMC from SHR and WKY rats using specific phosphotyrosine antibodies. As shown in “Fig 10“, phospho-specific-Tyr^1173^-EGF-R (A), and phospho-specific-Tyr^857^-PDGF-R (B) detected a single band at 170 and 190 kDa corresponding to EGF-R and PDGF-R, respectively, in VSMC from both SHR and WKY rats. However, the extent of EGF-R and PDGF-R phosphorylation was greater by about 65% and 110%, respectively, in VSMC from SHR compared with VSMC from WKY rats and SNAP treatment attenuated the increased phosphorylation of EGF-R and PDGF-R to almost WKY control levels. In addition, this treatment also attenuated the phosphorylation of EGF-R and PDGF-R by about 20% and 30% respectively in VSMC from WKY rats. In addition, the expression of total EGF-R and PDGF-R was not altered in VSMC from SHR compared to WKY rats and also by SNAP treatment.

### SNAP decreases enhanced activation of c-Src in VSMC from SHR

**Fig 11:**
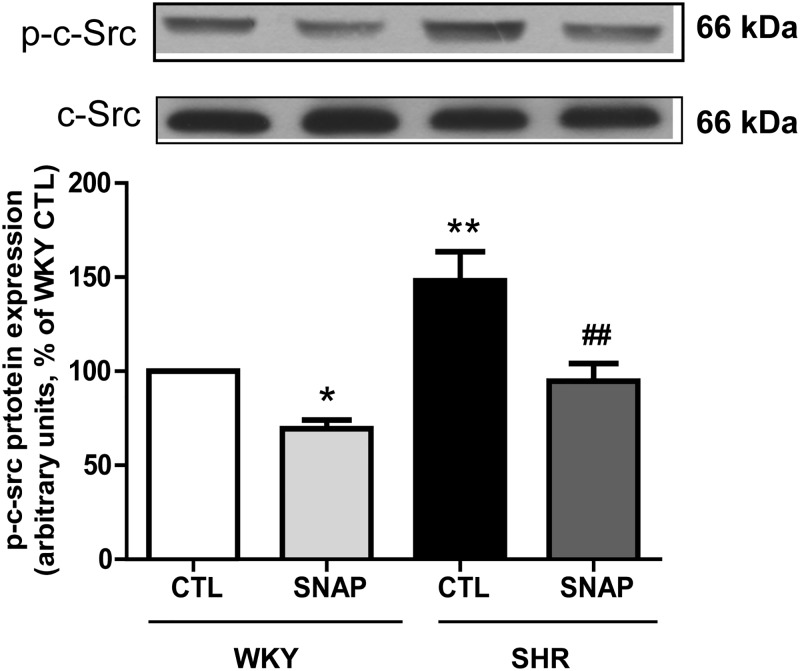
Effect of SNAP on expression c-Src phosphorylation in VSMC from 12-week old SHR and age-matched WKY rats. Confluent VSMC were incubated in the absence (control) or presence of SNAP (100 μM) for 24 h. The cell lysates were prepared and subjected to Western blot analysis using specific antibodies against phosphorylated c-Src (upper panels) as described in the Materials and methods. Quantification of protein bands was done by densitometric scanning (lower panels). The results are expressed as a percentage of the WKY control (CTL), taken as 100%. Values are the mean ± SD of 4 separate experiments using different cell populations from different animals. *P<0.05, ** P<0.01 vs WKY CTL group, ^##^ P<0.001 vs SHR CTL group.

We and others have shown earlier that c-Src, an upstream signaling molecule contributes to the activation of EGF-R [24, 50], and since SNAP attenuated the activation of EGFR and PDGFR, it was of interest to examine if SNAP that could also attenuate the enhanced activation of c-Src in VSMC from SHR. To test this, the effect of SNAP on the phosphorylation of c-Src was assessed in VSMC from SHR and WKY rats. Results shown in “Fig 11“indicate that the phosphorylation of Tyr^418^ on c-Src was significantly augmented by about 50% in VSMC from SHR compared with WKY rats and SNAP attenuated the enhanced phosphorylation of c-Src to WKY levels. In addition, the expression of total c-Src was not different in control and SNAP-treated VSMC from SHR and WKY rats.

### SNAP attenuates hyperproliferation of VSMC from SHR

**Fig 12:**
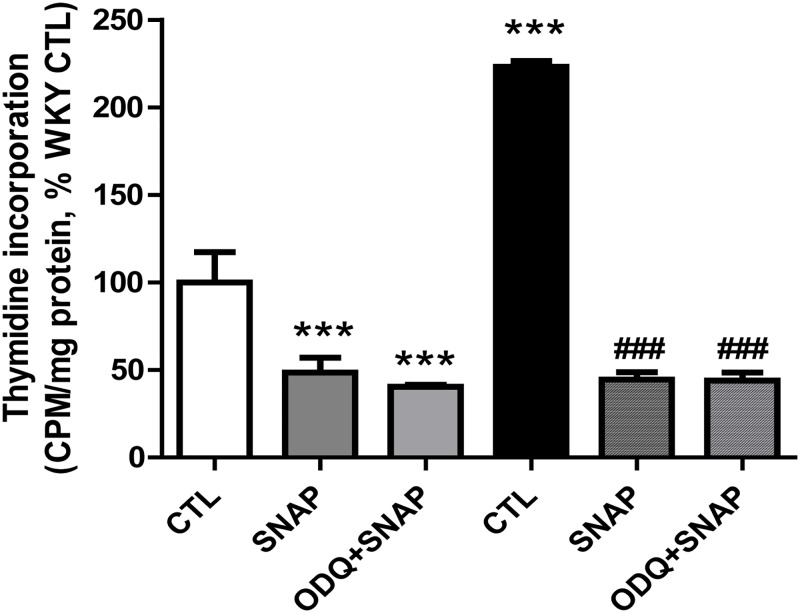
Effect of SNAP on DNA synthesis in VSMC from 12 week-old SHR and age-matched WKY rats. Confluent VSMC were incubated in the absence (control) or presence of 1H-(1, 2, 4) oxadiazolo [4, 3-a] quinoxalin-1-one (ODQ), for 30 mins prior to the treatment with SNAP for 24 h and thymidine incorporation was determined as described in Materials and methods. Results are expressed as % of WKY control (CTL), taken as 100%. Values are means ± SD of 3 separate experiments using different cell populations from different animals. *** P<0.001 vs WKY CTL group, ### P<0.001 vs SHR CTL group.

We earlier showed the implication of enhanced expression of Giα proteins in hyperproliferation of VSMC from SHR [10, 26]. Since SNAP decreases the enhanced expression of Giα proteins in VSMC from SHR, it was of interest to investigate if SNAP could also decrease the enhanced proliferation of VSMC of SHR. To test this, we examined the effect of SNAP on proliferation of VSMC from SHR and WKY rats, and the results are shown in “Fig 12“. As reported previously [10], VSMC from SHR exhibited enhanced proliferation (∼130%) compared with WKY as determined by [^3^H] thymidine incorporation which was completely attenuated by SNAP treatment. In addition, SNAP also decreased the proliferation of VSMC from WKY rats by about 50%. Furthermore, SNAP-induced attenuation of proliferation of VSMC was not reversed by ODQ and suggests that SNAP decreased the proliferation by cGMP-independent mechanism.

## Discussion

We showed earlier the implication of enhanced levels of endogenous Ang II and ET-1 and oxidative stress in enhanced expression of Giα proteins and exaggerated cell growth in VSMC from SHR as compared to WKY rats [10, 24, 25]. Furthermore, the inhibition of NO synthase by *N*^ω^-nitroarginine methyl ester (L-NAME) that decreases the intracellular levels of NO has been shown to increase the levels of Giα proteins and blood pressure in rats [17]. However, in the present study, we report for the first time that the SNAP-induced augmentation of intracellular levels of NO that were decreased in VSMC of SHR [33] attenuates the overexpression of Giα proteins and enhanced proliferation through the inhibition of ROS and ROS-mediated c-Src signaling pathways and suggest the implication of decreased levels of intracellular NO in enhanced expression of Giα proteins and hyperproliferation of VSMC from SHR.

We showed that both NO donors SNAP and SNP also attenuated the enhanced expression of Giα proteins in VSMC from SHR. However, the fact that ODQ, an inhibitor of soluble guanylyl cyclase that inhibited SNAP-induced intracellular levels of cGMP, was unable to reverse the attenuated expression of Giα protein induced by SNAP, suggest that SNAP-evoked attenuation of overexpression of Giα proteins is not mediated through a cGMP-dependent mechanism and may involve some other mechanisms. In this regard, NO-mediated inhibition of pulmonary microvascular smooth muscle cell proliferation was reported to involve cyclin-dependent kinase inhibitor P^21^ and not cGMP [51]. In addition, NO-mediated stimulation of K_ATP_ channels was also shown to be mediated through Ras and MAPK pathways and not through the cGMP signaling pathway [52].

Our results showing that 8-Br-cGMP, a cell permeable analog of cGMP and an intracellular second messenger for NO action also attenuated the enhanced expression of Giα proteins in VSMC from SHR at high concentration (0.5 mM), are consistent with earlier studies showing that treatment of A10 VSMC with 8-Br-cGMP attenuated the expression of Giα proteins [45, 46], Several studies have shown that intracellular levels of basal or SNP-stimulated cGMP in VSMC or vascular tissue from SHR are quite low in the range of fmoles to pmoles [53, 54]. We also report that SNAP increased the levels of intracellular cGMP in VSMC from SHR and WKY rats in pmoles. Taken together, it may be suggested that the intracellular levels of cGMP produced by SNAP are not high enough to exert its inhibitory effect on Giα protein expression. This notion is substantiated by the study showing that 8-BrcGMP at lower concentrations than 0.5 mM was unable to inhibit the enhanced expression of Giα proteins (unpublished observations).

We also report that treatment of VSMC from SHR with SNAP attenuates the enhanced proliferation of VSMC from SHR towards control levels by cGMP-independent pathway because ODQ was unable to reverse the SNAP-induced antiproliferative effect. Our results are in agreement with the studies of Lahteenmaki [54] who also showed that NO and NO donors like SNAP inhibited VSMC proliferation by a cGMP-independent mechanism. The role of Giα proteins in the regulation of cell proliferation has also been shown [11, 12]. We recently reported the implication of enhanced expression of Giα proteins in hyperproliferation of aortic VSMC from SHR [26]. In addition, C-ANP_4-23_ treatment that attenuates the enhanced expression of Giα proteins was also shown to inhibit the enhanced proliferation of aortic VSMC from SHR [55] Taken together, it is suggested that SNAP-induced attenuation of enhanced expression of Giα proteins may be the contributing factor in its antiproliferative effect.

The implication of MAP kinase signaling in enhanced expression of Giα-2 and Giα-3 protein and cell proliferation induced by vasoactive peptides [9] and in SHR [10, 24, 25, 39, 56] is well documented. In the present study, we show that SNAP-induced inhibition of enhanced expression of Giα proteins is also mediated through MAP kinase signaling because the inhibition of MAP kinase activity by pretreatment of cells with PD98059, a MEK inhibitor prevented SNAP to inhibit the enhanced expression of Giα proteins to control levels. In addition, the fact that SNAP also inhibits the enhanced phosphorylation of ERK1/2 in VSMC from SHR further suggests that SNAP decreases the expression of Giα proteins and hyperproliferation of VSMC through MAP kinase signaling.

We earlier showed the role of growth factor receptor activation in enhanced expression of Giα proteins and hyperproliferation of VSMC from SHR [10, 24, 25]. Our results showing that SNAP attenuates the enhanced phosphorylation of EGF-R and PDGF-R in VSMC from SHR further suggest that SNAP-induced attenuation of enhanced expression of Giα proteins may also be mediated through the inhibition of growth factors receptor activation.

We demonstrate that SNAP attenuates the enhanced expression of AT1 receptor, enhanced activity of NAD(P)H oxidase, enhanced levels of O_2_^-^, enhanced expression of, Nox4, and p47^phox^ p22 ^phox^ proteins as well as enhanced levels of TBARS and protein carbonyls in VSMC from SHR towards control WKY levels and suggest that SNAP-induced attenuation of enhanced expression of Giα proteins may be attributed to its ability to decrease the enhanced levels of AT1 receptor and AT1 receptor-induced oxidative stress. In this regard, enhanced oxidative stress due to the augmented levels of endogenous Ang II and ET-1 through the transactivation of growth factor receptors has been reported to contribute to the enhanced expression of Giα proteins in VSMC from SHR [25, 34, 39, 56]. In addition, the inhibition of intracellular levels of NO by L-NAME treatment of rats has been shown to increase the expression of Giα proteins and high blood pressure through AT1 receptor because losartan, AT1 receptor antagonist attenuated the enhanced expression of Giα proteins and high blood pressure in these rats [57]. Furthermore, NO-mediated downregulation of AT1 receptor [58] and AT1 receptor-induced migration of aortic VSMC has also been reported [59]. The downregulation of AT1 receptor induced by NO donors was shown to be cGMP-independent [58]. However, the mechanism by which NO inhibits AT1 receptor expression in VSMC from SHR is not clear and needs to be investigated,

Oxidative stress through the activation of c-Src has been reported to transactivate growth factor receptor [10, 24, 25]. Our data demonstrating that SNAP attenuates the enhanced phosphorylation of c-Src to control levels, suggest that SNAP-induced inhibition of c-Src activation may contribute to the inhibition of the activation of downstream signaling molecules and thereby results in the attenuation of enhanced expression of Giα proteins and hyperproliferation. These results suggest that the enhanced expression of Giα proteins, AT1 receptor as well as EGFR and IGF-1R and other signaling molecules may reflect a phenotype switch of VSMC from a contractile state to a synthetic state in SHR. In this regard, VSMC profile conversion has been shown to be associated with the modulation of protein expression of certain membrane molecules implicated in VSMC hypertrophy, proliferation and contractility [60, 61].

**Fig 13:**
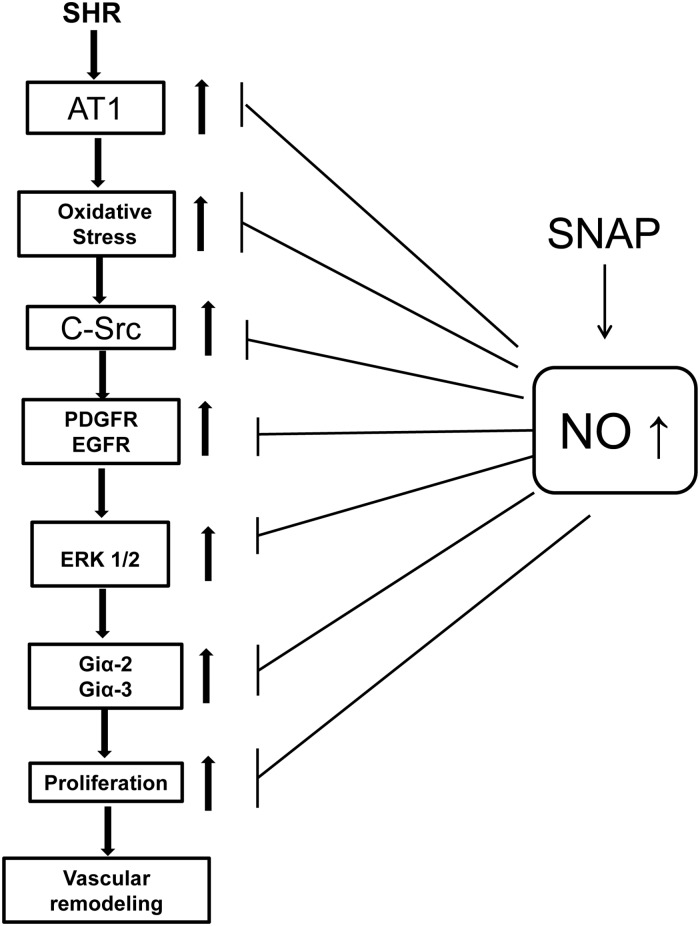
Schematic diagram summarizing the possible mechanisms by which SNAP attenuates the enhanced expression of Giα proteins and hyperproliferation of VSMC.

In conclusion, we have shown that the augmentation of the intracellular levels of NO by SNAP attenuates the enhanced expression of AT1 receptor and all the downstream signaling molecules including oxidative stress, activation of c-Src and growth factor receptors, MAPK signaling, that were shown to be implicated in enhanced expression of Giα proteins and hyperproliferation [10, 24, 25] (“Fig 13“). Thus, it may be suggested that the inhibition of the enhanced expression of AT1 receptor, enhanced oxidative stress and downstream signaling pathways induced by SNAP may contribute to the attenuation of increased expression of Giα proteins and thereby hyperproliferation of VSMC from SHR and that elevating the intracellular levels of NO in SHR may have protective effect against oxidative stress-induced vascular complications of hypertension.

## Abbreviations

8-Br-cGMP: 8-Bromoguanosine 3`,5`-cyclicmonophosphate
DOCA: deoxycorticosterone acetate
EGF-R: epidermal growth factor receptor
L-NAME: N^ω^-nitro-L-arginine methyl ester
ODQ: 1H (1, 2, 4) oxadiazole (4, 3-a) quinoxalin-1-one
PDGF-R: platelet-derived growth factor receptors
SHR: spontaneously hypertensive rats
SNAP: S-Nitroso-N-acetyl-DL-penicillamine
SNP: sodium nitroprusside
TBARS: Thiobarbituric acid-reactive substances
VSMC: Vascular smooth muscle cells
